# Optimizing Disease Outbreak Forecast Ensembles

**DOI:** 10.3201/eid3009.240026

**Published:** 2024-09

**Authors:** Spencer J. Fox, Minsu Kim, Lauren Ancel Meyers, Nicholas G. Reich, Evan L. Ray

**Affiliations:** University of Georgia, Athens, Georgia, USA (S.J. Fox);; University of Massachusetts Amherst, Amherst, Massachusetts, USA (M. Kim, N.G. Reich, E.L. Ray);; University of Texas at Austin, Austin, Texas, USA (L.A. Meyers);; Dell Medical School, Austin (L.A. Meyers); Santa Fe Institute, Santa Fe, New Mexico, USA (L.A. Meyers)

**Keywords:** Infectious disease forecasting, ensemble forecasting, outbreak, disease models, viruses, COVID-19, influenza, hospitalizations, United States

## Abstract

On the basis of historical influenza and COVID-19 forecasts, we found that more than 3 forecast models are needed to ensure robust ensemble accuracy. Additional models can improve ensemble performance, but with diminishing accuracy returns. This understanding will assist with the design of current and future collaborative infectious disease forecasting efforts.

Real-time collaborative forecast efforts have become the standard to generate and evaluate forecasts for infectious disease outbreaks (*1*,*2*). Individual forecasts are aggregated into an ensemble prediction that has historically outperformed individual models and is the primary external communication used (*3*–*5*). Because of the focus on the singular ensemble model and the costs associated with producing individual forecasts, public health officials starting or maintaining a forecast hub face 2 key challenges: identifying target participation rates and optimizing ensemble performance of participating models. To guide this decision-making, we analyzed data from recent US-based collaborative outbreak forecast hubs to identify how the size and composition of an ensemble influences performance.

We analyzed hub forecasts for influenza-like illness (ILI) from 2010–2017 (*5*); for COVID-19 reported cases, hospital admissions, and deaths from 2020–2023 (*6*); and for influenza hospital admissions from 2021–2023 (*7*). For each hub, we identified time periods with maximal model participation that had at least 2 increasing and 2 decreasing epidemiologic phases and obtained forecasts for individual models that produced >90% of all possible forecasts (Appendix Table 1, Figure 1). For each ensemble size, *n_D_* ∈ {1, … ,*N_D_*}, where *N_D_* is the disease-specific total number of models matching our inclusion criteria; we created unweighted ensemble forecasts for every combination of individual models of size *n_D_*. We followed the hub forecast methodologies and made probabilistic forecasts for ILI by using a linear pool methodology (*5*), and we made quantile forecasts for all others by taking the median across all individual forecasts (Figure 1) (*8*). For each hub, we compared the ensemble performance against 2 hub-produced models. The first is a baseline model that produces naive forecasts and serves as a skill reference point; and the second is the published ensemble produced in real-time that is an unweighted ensemble of all submitted forecasts and is the current standard for performance (*3*,*5*). We summarized probabilistic ensemble forecast skill by using the log score for ILI forecasts and the weighted interval score for all others (*9*,*10*). We took the reciprocal of the log score so that lower values would indicate better performance similar to the weighted interval score (Appendix).

**Figure 1:**
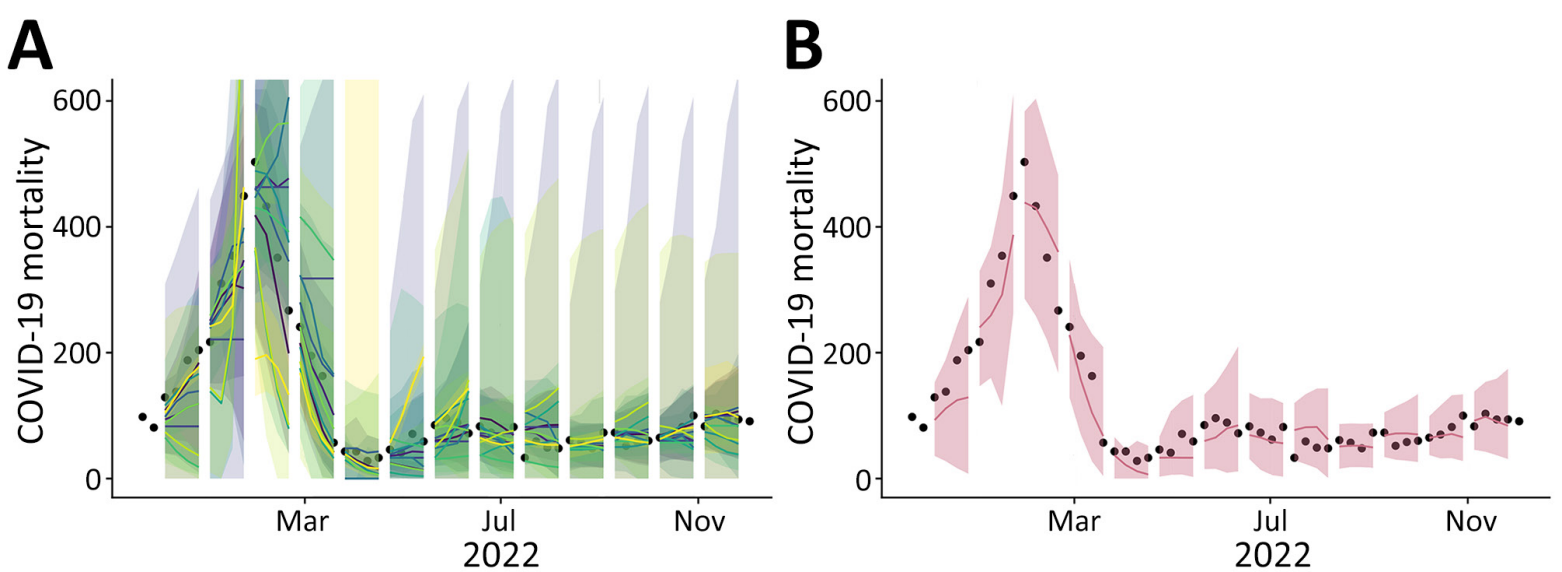
Comparison between individual and ensemble forecasts for COVID-19 mortality for Massachusetts, USA, from 1–4 weeks ahead, November 15, 2021–December 3, 2022, in study of optimizing disease outbreak forecasting ensembles. A) Individual forecasts of 10 models meeting inclusion criteria compared with weekly COVID-19 mortality estimates. B) An ensemble forecast constructed by taking the median across 10 individual forecasts compared with weekly COVID-19 mortality estimates. Black dots, weekly COVID-19 mortality estimates; colored lines, medians; ribbons, 95% prediction intervals.

Looking across all ensemble sizes and combinations, we found that including more models improved average forecast performance and that all ensembles composed of >3 models outperformed the baseline model (Figure 2). Further increases to the ensemble size slightly improved the average forecast performance, but substantially decreased the variability of performance across ensembles. When we increased the ensemble size of influenza hospital admission forecasts from 4 to 7, the average performance improved by 2%, but the interquartile range decreased by 56.5%. Increasing the ensemble size therefore reduces the variability in expected performance of an ensemble.

**Figure 2:**
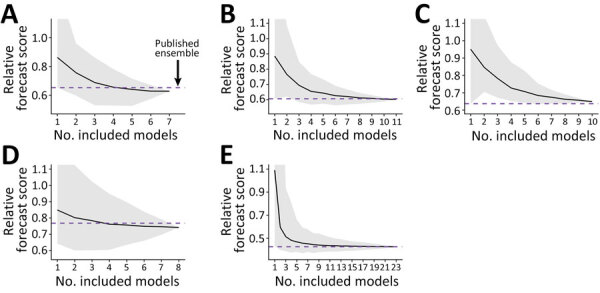
Summarized ensemble forecast scores from collaborative forecast efforts in study of optimizing disease outbreak forecasting ensembles. Scores correspond to the average forecast performance during testing periods across all dates, locations, and forecast horizons (Appendix Table 1). All scores are standardized by the baseline forecast model for that metric (Y = 1). Scores <1 indicate better accuracy than baseline. A) COVID-19 cases with 15 included models. B) COVID-19 admissions with 17 included models. C) COVID-19 deaths with 19 included models. D) Influenza admissions with 21 included models. D) Influenza-like illness with 23 included models. Solid black lines indicate mean scores; gray shading indicates minimum–maximum range. Horizontal purple dashed line indicates unweighted published ensemble used as standard.

To assist with decision-making regarding optimal ensemble assembly, we tested 2 approaches for model selection on the basis of past performance. We either ranked models by their individual performance and chose the top *n_D_* models (individual rank) or we compared the performance of all ensemble combinations of size *n_D_* and chose the models from the top performing ensemble (ensemble rank). Across all hubs, the individual rank methodology outperformed randomly assembled ensembles of the same size 63% (range 33.1%–87.2%) of the time, and the ensemble rank methodology outperformed randomly assembled ensembles of the same size 87.9% (range 70.9%–99.7%) of the time (Appendix Table 2, Figure 2). Performance of those ensembles is similar during both the training and testing periods, suggesting that ensemble performance is consistent through time (Appendix Figures 2, 3). Overall, ensemble rank outperforms individual rank for ensemble construction for 89.8% (range 66.7%–100%) of all sizes, and it provides a 6.1% (range 1.3%–11.9%) skill improvement (Appendix Table 2). The size 4 ensemble rank performed similarly to the published hub ensemble, although performance often declined with additional models (Appendix Figures 2, 3). Relative forecast performance across ensemble strategies was consistent when stratified by the ensemble size, forecast location, forecast date and phase of the epidemic, forecast target, and the skill metric (Appendix Figures 4–18).

Our results provide guidance for future collaborative forecast efforts. Hub organizers should target a minimum of 4 validated forecast models to ensure robust performance compared with baseline models. Adding more models reduces the variability in expected ensemble performance but might come with diminishing returns in average forecast skill. Organizers should use past ensemble performance rather than individual performance when selecting models to include in forecast ensembles; it is likely that further gains and different relationships between ensemble size and performance will come from weighted ensemble approaches (*8*). As public health officials and researchers look to expand collaborative forecast efforts, and as funding agencies allocate budgets across methodological and applied forecast efforts, our results can be used to identify target participation rates, assemble appropriate forecast models, and further improve ensemble forecast performance.

Appendix Additional information about optimizing disease outbreak forecasting ensembles.
