# Supplementary material for: Optimizing Disease Outbreak Forecast Ensembles
Source: Emerg Infect Dis. 2024 Sep;30(9):1967–9. doi: 10.3201/eid3009.240026 (PMC11347000; doi:10.3201/eid3009.240026)
Supplement: Appendix — Additional information about optimizing disease outbreak forecasting ensembles. [file 24-0026-Techapp-s1.pdf]

Article DOI: https://doi.org/10.3201/eid3009.240026

EID cannot ensure accessibility for supplementary materials supplied by authors. Readers who have difficulty accessing supplementary content should contact the authors for assistance.

Optimizing Disease Outbreak Forecast Ensembles

Appendix

Methodological overview

The following sections describe our approach to (1) choosing the models and time periods to include for each forecast effort (2); constructing ensemble forecasts for the selected retrospective time periods (3); creating and scoring ensemble forecasts for random subsets of available individual models; and (4) constructing the individual rank and ensemble rank ensembles with selected component models.

Collaborative forecast competitions investigated

To ensure our ensemble analysis results were consistent and robust, we carried out the ensemble analysis on all United States-based collaborative forecast efforts with publicly available submission formats (i.e., those that organized submissions using a hub format on Github). These include the coordinated efforts to forecast influenza-like illness percent over seven influenza seasons (*1*), influenza hospitalizations beginning in 2021 (*2*), and COVID-19 case counts, hospital admissions, and mortality beginning in 2020 (*3*–*5*).

Model and time period selection

To carry out the analysis we selected time periods for each collaborative forecast effort that had the maximum number of individual component forecast models with submissions for at least 90% of all possible forecasts. We included the baseline forecast models and we excluded all ensembles of forecasts from other models that contributed to each competition as that follows the inclusion criteria for the current Published ensemble model. We also included the Published ensemble for each competition to be used as a reference for comparison, but these models were not incorporated as possible members of the ensembles that we created. We split up the time periods into training and testing periods, ensuring that each period for each metric had at least a period of epidemic growth and decline (Appendix Figure 10-S14). For all of the hubs except for

COVID-19 hospital admissions, forecast participation dropped significantly over time, limiting the total number of models that could be used in the analysis (Appendix Figure 1). The final time periods included models, and specified baseline models can be found in Appendix Table 1.

Ensemble creation, forecasting, and scoring

For all but the multiyear ILI % competition, we created ensembles for all possible combinations of individual models of a specified size, n_D, for all values of n_D from 1 to N_D where N_D is the total number of models included for that forecasting exercise (Appendix Table 1). This yielded 127, 2047, 1023, and 255 total ensembles for the COVID-19 case counts, COVID-19 hospital admissions, COVID-19 mortality counts, and influenza hospital admissions respectively. For these competitions, forecasts were submitted in a quantile format, meaning that for every forecast target (e.g., COVID-19 admission counts for Illinois at a 1-week horizon made on a specific date), forecast models provided their prediction distribution with a set of 23 quantiles at probability levels 0.01, 0.025, 0.05, 0.10, 0.15, …, 0.95, 0.975, 0.99 (*4*). We followed the methodology used to create real-time, published forecast ensembles in (*4*) and created an unweighted ensemble forecast by taking the median for each probability level across all of the included individual models for each forecast location, date, and target using the hubEnsembles R package (*6*). As not every model submitted forecasts for every date and horizon, some ensemble forecasts of a specified size, n_D, had fewer than n_D component models. We did not exclude these from our analysis, because it was a rare occurrence, and it replicates the real world scenario where some models will miss some submissions.

We scored all forecasts for all ensemble models following the scoring methods from (*4*) using the methodologies made available in the covidHubUtils R package (*7*). We focused our analysis on the weighted interval score (WIS), which captures overall forecast performance, and the prediction interval coverage (PIC), which estimates the calibration of forecast uncertainty (*7*–*9*). WIS is a proper score that evaluates the difference between a predictive distribution provided in quantile interval format and the true observations (*8*) and can be calculated by aggregating scores for all prediction intervals as:

$$\mathrm{IS}_{\alpha}(\mathrm{F},\mathrm{y}) = (\mathrm{u}-\mathrm{l}) + \frac{2}{\alpha} * (\mathrm{l}-\mathrm{y}) * \mathbf{1}(\mathrm{y}<\mathrm{l}) + \frac{2}{\alpha} * (\mathrm{y}-\mathrm{u}) * \mathbf{1}(\mathrm{y}>\mathrm{u})$$

$$\mathrm{WIS}_{\alpha_{0:K}}(F, y) = \frac{1}{K + \frac{1}{2}} * \left(w_0 * |y - m| + \sum_{k=1}^{K} \{ w_k * \mathrm{IS}_{\alpha_k}(F, y) \} \right)$$

Where *F* is the predictive distribution, *y* is the true observation, $(1 - \alpha) \times 100\%$, is a single interval width, *u* and l indicate the $\frac{\alpha}{2}$ and $1 - \frac{\alpha}{2}$ quantiles of *F* respectively, $\mathbf{1}(y < l)$ is an indicator that equals 1 if $y < l$ and 0 otherwise, $\mathbf{1}(y > u)$ is an indicator that equals 1 if $y > u$ and 0 otherwise, *K* is the number of intervals being evaluated (11 in our case), *m* is the median prediction, $w_0 = \frac{1}{2}$, and $w_k = \frac{\alpha_k}{2}$. As defined, WIS can be interpreted as a weighted sum of the 11 interval scores for each forecast, and is equivalent to absolute error if only point forecasts are evaluated.

PIC evaluates model uncertainty calibration and is defined as the probability that a specified prediction interval captures the true observed value. We compute the PIC for the $(1 - \alpha) \times 100\%$ prediction interval as:

$$\mathrm{PIC} = \frac{1}{N_f} \sum_{i=1}^{N_f} \mathbf{1}\left(l_{\alpha,i} \le y_i \le u_{\alpha,i}\right)$$

where N_f is the number of forecasts or observations being evaluated and $\mathbf{1}\left(l_{\alpha,i} \le y_i \le u_{\alpha,i}\right)$ is an indicator function that equals 1 if $l_{\alpha,i} \le y_i \le u_{\alpha,i}$. For a well calibrated model one expects that PIC = 0.95 for the 95% prediction interval, though it is common to obtain lower PIC values than expected. While PIC is not a proper score, it is a measure of reliability that is used to assess model calibration and contributes to public health decisions regarding whether forecasts should be distributed (*9*). Following the methods in (*4*), we analyzed the average WIS and PIC for only forecasts of the states and territories, as national-level forecasts can skew forecast performance estimates. For these forecasting exercises, the Published ensemble was the ensemble produced by the hub using all available models (including those that did not meet our eligibility criteria); thus, the Published ensemble generally included more models than the ensembles that we considered.

For the multiyear ILI % analysis, we followed a different creation and scoring methodology due to the different forecast format and the number of individual component models. For this competition, teams were asked to submit 100% of all forecast targets and dates

retrospectively, so we included all 23 individual component models that were successfully submitted on GitHub (*10*). Given the 23 models, there would be 8,388,607 total possible ensemble combinations, which was computationally impractical to run, score, and post-process. For ensembles of size n_D that had fewer than 1,000 possible combinations, we ran all of those combinations, but for those that had more than 1,000 possible combinations, we randomly selected 1,000 ensembles from the options. In total, we included 18,553 total ensemble combinations in the analysis and we used the largest ensemble of size 23 as the Published ensemble.

Forecasts for this competition were submitted in a bin probability format rather than an interval format. As described in the FluSight papers (*1*,*11*,*12*), models provided predicted probabilities for pre-defined bins for each forecast target. For forecasting the onset and peak week, bins corresponded to single weeks, though there was an additional onset week bin that corresponded to a scenario where the influenza season onset definition was not met during that year. For the ILI peak and week ahead forecasts, 11 bins were used, each covering a 1% range from 0% to 10%, with a final bin corresponding to values greater than 10%. Forecast models assigned probabilities that the true observation would fall within each bin, thus capturing a discrete probability distribution.

We produced ensemble forecasts for all ensemble combinations, targets, and dates using the ensemble methodology used in (*13*). Specifically, for every combination of individual models, we created a linear pool ensemble model that assumes equal weights across all included component models for all forecast dates and targets. To describe this method, we denote the bin endpoints by (b_0, b_1, …, b_K), where K is the total number of bins. A prediction for a particular target and date from model m consists of an assignment of predictive probabilities $p_{m,k}$ to each bin (b_{k-1}, b_k], with the sum of the bin probabilities equal to 1: $\sum_{k=1}^{K} p_{m,k} = 1$. The ensemble prediction for each bin is computed as the mean of the component model predictions; i.e., if there are M models in total, the probability assigned to the bin (b_{k-1}, b_k], by the ensemble is $p_{ensemble,k} = \frac{1}{M}\sum_{m=1}^{M} p_{m,k}$. To compute these ensemble predictions, we used publicly available code provided in (*14*).

We followed the scoring methodology of (*1*) and used the FluSight R package functions to score all ensemble forecasts (*15*). Specifically, we evaluated forecasts using the log score,

which is a proper score that captures forecast accuracy and precision (*16*). The log score for a probabilistic forecast is defined as $\ln\big(\mathrm{f}(\mathrm{y}|\mathrm{x})\big)$, where $\mathrm{f}(\hat{\mathrm{y}}|\mathrm{x})$ is the predicted probability distribution for the forecast on data x, y is the observed observation, and *ln* indicates the natural logarithm. We assign score values of -10 to any values less than -10 similar to (*1*). Following the methodology described in (*13*) we summarized the individual forecast scores for every date and target as $z = e^{\mathrm{mean}(ls)}$, where z is the resulting summary forecast score and ls is the log score calculated as described above. We took $\frac{1}{z}$ as the final measure of forecast skill, so that smaller values indicated better forecast performance consistent with the interpretation of WIS.

Ensemble methodology

In the text we present results from three different ensemble methodologies

1. A random sampling methodology (Random) that presents the forecast scores for ensembles of size *n* across all created ensembles of that size in the testing period. Since we create all possible combinations of ensembles for all but the multiyear ILI % analysis, presenting these results is equivalent to presenting range and average results if one were to randomly combine models to achieve a specified ensemble size. For the multiyear ILI % analysis, in settings where the number of created ensembles was capped at 1,000 it presents the range and mean of scores for the ensembles of randomly selected individual models.

2. A component model selection scheme that relies on the individual rank of the component models from the training period (Individual rank). To create an ensemble of size *n*, we choose the top *n* individually performing models from the training period to use as the members of the ensemble in the testing period. Results from these ensembles are only presented in the testing period.

3. A component model selection scheme that relies on the ensemble rank of the investigated ensemble models from the training period (Ensemble rank). For this model, we create an ensemble of a specified size *n* by identifying the ensemble of size *n* that had the best forecast performance in the training period. An ensemble using those same component models was then used to generate forecasts for the testing period. For the multiyear ILI % analysis, not all ensemble combinations were created and analyzed in the training period (as described in the previous

section), so we limited our ensemble choice to only those that were analyzed from the random sample created. This means we may not have chosen the best performing ensemble from the training period across all possible options.

Baseline forecast models

As described above and within the main text, we included the original hub-published baseline forecast models to use as a forecast skill reference point for each of the individual forecast hub results, since it is difficult to interpret the absolute value of the forecast skill metrics. The ILI % forecast hub used a seasonal baseline model, while all other forecast hubs used a flatline forecaster.

The seasonal baseline model used in the multiyear ILI % forecast hub (ReichLab_kde) was described in (*1*), and uses a kernel density estimation procedure for seasonal target forecasts and a generalized additive model spline for weekly incidence forecasts. Since both of these methods solely use historical influenza season data to produce forecasts, it serves as a reasonable baseline model for seasonal outbreaks. Code for the model can be found on GitHub (https://github.com/reichlab/2017-2018-cdc-flu-contest/blob/dcb99465bccbe1167e196878182b2e84749b6d87/R/kde-utils.R). For all other forecast hubs, the flatline baseline model described in (*4*) was used. In short, the model makes median predictions assuming there will be no change to the most recent observation (producing a flatline into the future), and the quantile predictions around the median are drawn from the first differences of the time-series for the specific region of interest. The model produces symmetric forecast quantiles by combining the first difference and the negative first difference distribution, and sampling from a smoother version of the resulting symmetrized distribution. The final quantile forecasts are truncated to ensure no negative numbers. The flatline baseline forecast model is similar to a truncated random walk time-series model for each specific location. Code for the flatline baseline model can be found here: https://github.com/reichlab/simplets.

Data and code availability

All forecasts and ground truth data used in the analysis are publicly available in their specific forecast repositories. Code that gathers the data from the individual competitions and replicates the analysis presented in this manuscript are available (https://github.com/sjfox/ensemble-size).

References

1. Reich NG, Brooks LC, Fox SJ, Kandula S, McGowan CJ, Moore E, et al. A collaborative multiyear, multimodel assessment of seasonal influenza forecasting in the United States. Proc Natl Acad Sci U S A. 2019;116:3146–54. PubMed https://doi.org/10.1073/pnas.1812594116

2. Flusight-forecast-data. Github; [cited 2023 Jul 12]. https://github.com/cdcepi/Flusight-forecast-data

3. Cramer EY, Huang Y, Wang Y, Ray EL, Cornell M, Bracher J, et al.; US COVID-19 Forecast Hub Consortium. The United States COVID-19 forecast hub dataset. Sci Data. 2022;9:462. PubMed https://doi.org/10.1038/s41597-022-01517-w

4. Cramer EY, Ray EL, Lopez VK, Bracher J, Brennen A, Castro Rivadeneira AJ, et al. Evaluation of individual and ensemble probabilistic forecasts of COVID-19 mortality in the United States. Proc Natl Acad Sci U S A. 2022;119:e2113561119. PubMed https://doi.org/10.1073/pnas.2113561119

5. COVID 19 forecast hub. [cited 2022 May 5]. https://covid19forecasthub.org

6. GitHub - reichlab/hubEnsembles: code for building ensembles for predictive modeling hubs. [cited 2023 Sep 6]. https://github.com/reichlab/hubEnsembles

7. GitHub - reichlab/covidHubUtils: Utility functions for the COVID-19 forecast hub. [cited 2023 Sep 6]. https://github.com/reichlab/covidHubUtils

8. Bracher J, Ray EL, Gneiting T, Reich NG. Evaluating epidemic forecasts in an interval format. PLOS Comput Biol. 2021;17:e1008618. PubMed https://doi.org/10.1371/journal.pcbi.1008618

9. Pinson P. International Institute of Forecasters. 2021. On the predictability of COVID-19. [cited 2022 Mar 31]. https://forecasters.org/blog/2021/09/28/on-the-predictability-of-covid-19/

10. FluSightNetwork/cdc-flusight-ensemble. Component-models. [cited 2023 Sep 6]. https://github.com/FluSightNetwork/cdc-flusight-ensemble/tree/first-papers/model-forecasts/component-models

11. Biggerstaff M, Alper D, Dredze M, Fox S, Fung ICH, Hickmann KS, et al.; Influenza Forecasting Contest Working Group. Results from the centers for disease control and prevention's predict the 2013-2014 influenza season challenge. BMC Infect Dis. 2016;16:357. PubMed https://doi.org/10.1186/s12879-016-1669-x

12. Biggerstaff M, Johansson M, Alper D, Brooks LC, Chakraborty P, Farrow DC, et al. Results from the second year of a collaborative effort to forecast influenza seasons in the United States. Epidemics. 2018;24:26–33. PubMed https://doi.org/10.1016/j.epidem.2018.02.003

13. Reich NG, McGowan CJ, Yamana TK, Tushar A, Ray EL, Osthus D, et al. Accuracy of real-time multi-model ensemble forecasts for seasonal influenza in the U.S. PLOS Comput Biol. 2019;15:e1007486. PubMed https://doi.org/10.1371/journal.pcbi.1007486

14. FluSightNetwork/cdc-flusight-ensemble. stack_forecasts.R at v.2.0. [cited 2023 Sep 6] https://github.com/FluSightNetwork/cdc-flusight-ensemble/blob/v.2.0/scripts/stack_forecasts.R

15. An R package containing functions used in the CDC flu forecasting competition. [cited 2023 Sep 6]. https://github.com/jarad/FluSight

16. Gneiting T, Raftery AE. Strictly proper scoring rules, prediction, and estimation. J Am Stat Assoc. 2007;102:359–78. https://doi.org/10.1198/016214506000001437

Appendix Table 1. Selected time periods and models for each collaborative forecast effort. Baseline models for each metric are specified in bold. Only models that submitted at least 90% of forecasts during both the training and testing period were included (Appendix Figure 1).

Disease	Metric	Training period	Testing period	Included models
COVID-19	Cases	Nov 02, 2020 – Nov 08, 2021	Nov 15, 2021 – July 25, 2022	1. BPagano-RtDriven 2. CovidAnalytics-DELPHI 3. COVIDhub-baseline 4. CU-select 5. JHUAPL-Bucky 6. RobertWalraven-ESG 7. USC-SI_kJalpha
	Hospital admissions	Feb 07, 2022 – May 23, 2022	May 30, 2022 – Nov 07, 2022	1. BPagano-RtDriven 2. COVIDhub-baseline 3. CMU-TimeSeries 4. CU-select 5. CUB_PopCouncil-SLSTM 6. GT-DeepCOVID 7. Karlen-pypm 8. MOBS-GLEAM_COVID 9. MUNI-ARIMA 10. PSI-DICE 11. USC-SI_kJalpha
	Deaths	Nov 02, 2020 – Nov 08, 2021	Nov 15, 2021 – Nov 07, 2022	1. BPagano-RtDriven 2. COVIDhub-baseline 3. CU-select 4. GT-DeepCOVID 5. Karlen-pypm 6. MOBS-GLEAM_COVID 7. PSI-DRAFT 8. RobertWalraven-ESG 9. USC-SI_kJalpha 10. UCSD_NEU-DeepGLEAM
Influenza	Hospital admissions	Jan 10, 2022 –Jun 20, 2022	Oct 17, 2022 – April 03, 2023	1. CMU-TimeSeries 2. Flusight-baseline 3. GT-FluFNP 4. MOBS-GLEAM_FLUH 5. PSI-DICE 6. SGroup-RandomForest 7. SigSci-CREG 8. SigSci-TSENS
	Influenza-like illness (%)	Flu seasons: '2010/2011', '2011/2012', '2012/2013', '2013/2014'	Flu seasons: '2014/2015', '2015/2016', '2016/2017',	1. CU_EAKFC_SEIRS 2. CU_EAKFC_SIRS 3. CU_EKF_SEIRS 4. CU_EKF_SIRS 5. CU_RHF_SEIRS 6. CU_RHF_SIRS 7. CUBMA 8. Delphi_BasisRegression 9. Delphi_EmpiricalFutures 10. Delphi_ExtendedDeltaDensity 11. Delphi_MarkovianDeltaDensity 12. FluOutlook_Mech 13. FluOutlook_MechAug 14. FluX_ARLR 15. FluX_LSTM 16. LANL_DBMplus 17. Protea_Kudu 18. Protea_Springbok 19. ReichLab_kcde_backfill_none 20. ReichLab_kde 21. ReichLab_sarima_seasonal_difference_FALSE 22. ReichLab_sarima_seasonal_difference_TRUE 23. UA_EpiCos

Appendix Table 2. Forecast skill comparison for the Random, Ensemble rank, and Individual rank ensemble assembly methodologies. For Random assembly we calculated the probability that the ensemble or individual rank model matches or outperforms each of the produced ensemble combinations of the same size across all possible sizes. For comparisons of the ensemble rank and individual rank methods, probabilities are calculated as the proportion of all possible ensemble sizes where the average forecast score of the ensemble rank methodology matches or outperforms the average score of the individual rank method. In this comparison, we did not include results for comparing ensembles of size 1 or N_D because both methods choose the same ensemble.

Forecast variable	Probability individual rank matches or outperforms random choice	Probability ensemble rank matches or outperforms random choice	Probability ensemble rank matches or outperforms individual rank	Average skill improvement of ensemble vs individual rank (range)
COVID-19 cases	33.1%	70.9%	100.0%	11.9% (0.0%–22.6%)
COVID-19 admissions	87.2%	89.0%	66.7%	1.5% (−3.6%–14.5%)
COVID-19 deaths	64.1%	82.6%	87.5%	1.3% (−2.9%–5.7%)
Influenza admissions	71.8%	97.3%	100.0%	8.1% (1.7%–12.9%)
ILI %	61.8%	99.7%	95.2%	8.1% (0.0%–13.6%)

ILI, influenza-like illnesses

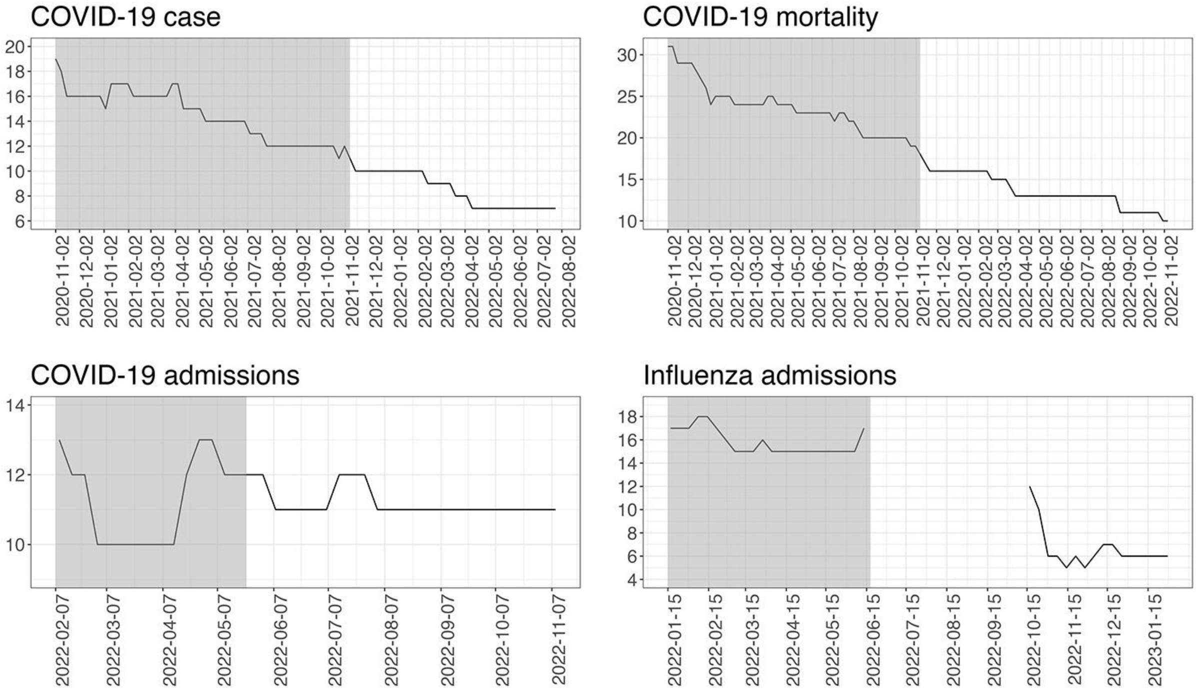

Appendix Figure 1. Model participation for the collaborative forecast hubs over time. Each plot indicates the total number of models that submitted at least 90% of all forecasts for the specified forecast date. Grey shaded regions indicate the testing period, while the remainder of the plot indicates the testing period. Time periods were chosen to maximize model participation, while also ensuring that each time period had at least an increasing and decreasing epidemic phase (Appendix Figure 10–S14).

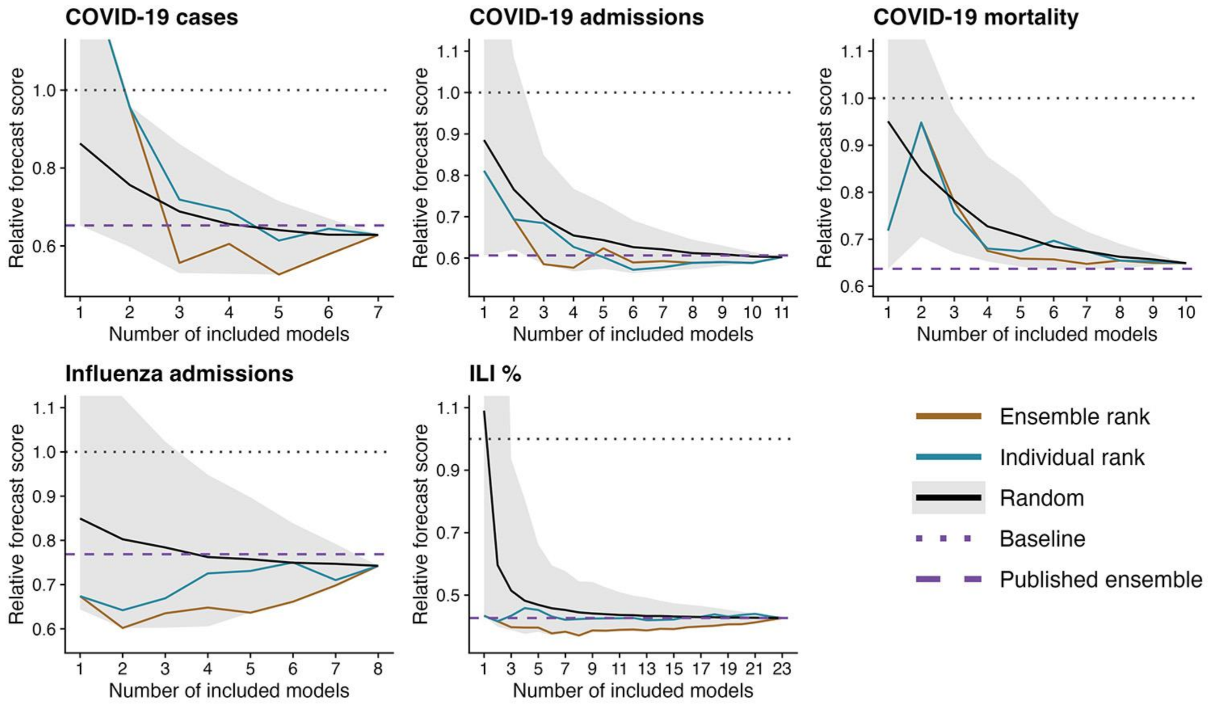

Appendix Figure 2. Forecast performance on recent influenza and COVID-19 collaborative forecast efforts comparing the number of models included in the ensemble and different ensemble methodologies. Summarized ensemble forecast scores from the collaborative forecast efforts for the weekly influenza-like illness (ILI) data provided by the CDC (ILI %), COVID-19 weekly case and mortality counts provided by JHU (COVID-19 cases and COVID-19 mortality), and COVID-19 and Influenza daily hospital admissions provided by HHS (COVID-19 admissions and Influenza admissions). Scores correspond to the average forecast performance during the respective testing periods across all dates, locations, and forecast horizons (Table S1). We plot the minimum (Grey region, lower), maximum (Grey region, upper), and mean (Solid black line) scores of random ensemble combinations of a given size (Random), and the trained ensembles composed of the top *n* individual performing models from the training period (Individual rank) or the best performing ensemble of size *n* from the training period (Ensemble rank). All scores are standardized by the baseline forecast model for that metric (horizontal dotted line), and the horizontal dashed line corresponds to the Published ensemble that is the unweighted ensemble across all models that submitted for a specific date and forecast target and is used as the gold-standard forecast prediction. Relative scores less than 1 indicate better accuracy than the Baseline. As the individual and ensemble rank methodologies do not attempt to optimize model weighting and simply make an unweighted ensemble, they reach a point where adding more models hurts forecast performance for most of the forecast hubs. On average across the testing phase, the Published ensemble included 15 models for COVID-19 cases, 17 models for COVID-19 admissions, 19 models for COVID-19 deaths, 21 models for influenza admissions, and 23 models for ILI.

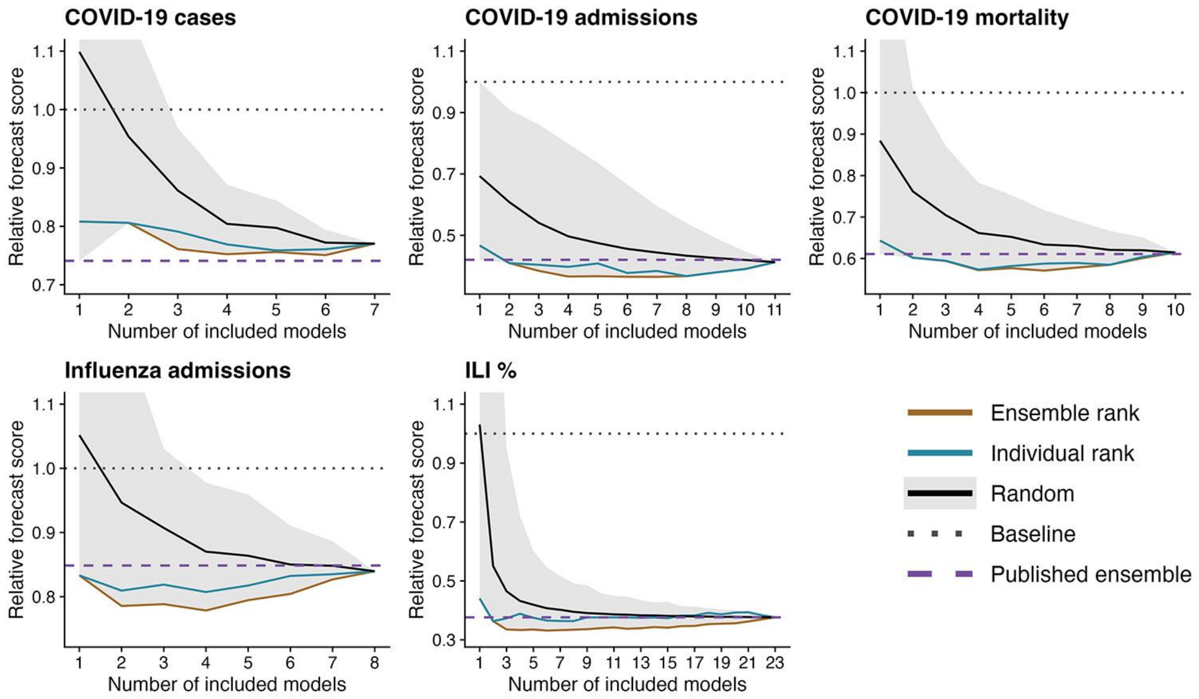

Appendix Figure 3. Forecast performance on the training period for recent influenza and COVID-19 collaborative forecast efforts comparing the number of models included in the ensemble and different ensemble methodologies. Summarized ensemble forecast scores from the collaborative forecast efforts for the weekly influenza-like illness (ILI) data provided by the CDC (ILI %), COVID-19 weekly case and mortality counts provided by JHU (COVID-19 cases and COVID-19 mortality), and COVID-19 and Influenza daily hospital admissions provided by HHS (COVID-19 admissions and Influenza admissions). Scores correspond to the average forecast performance during the respective training periods across all dates, locations, and forecast horizons (Table S1). We plot the minimum (Grey region, lower), maximum (Grey region, upper), and mean (Solid black line) scores of random ensemble combinations of a given size (Random), and the ensembles composed of the top n individual performing models from the training period (Individual rank) or the best performing ensemble of size n from the training period (Ensemble rank). These are included as a comparison with their ensemble performance in the testing period in the main manuscript. All scores are standardized by the baseline forecast model for that metric (horizontal dotted line), and the horizontal dashed line corresponds to the Published ensemble that is the unweighted ensemble across all models that submitted for a specific date and forecast target and is used as the gold-standard forecast prediction. Relative scores less than 1 indicate better accuracy than the Baseline. As the individual and ensemble rank methodologies do not attempt to optimize model weighting and simply make an unweighted ensemble, they reach a point where adding more models hurts forecast performance for most of the forecast hubs.

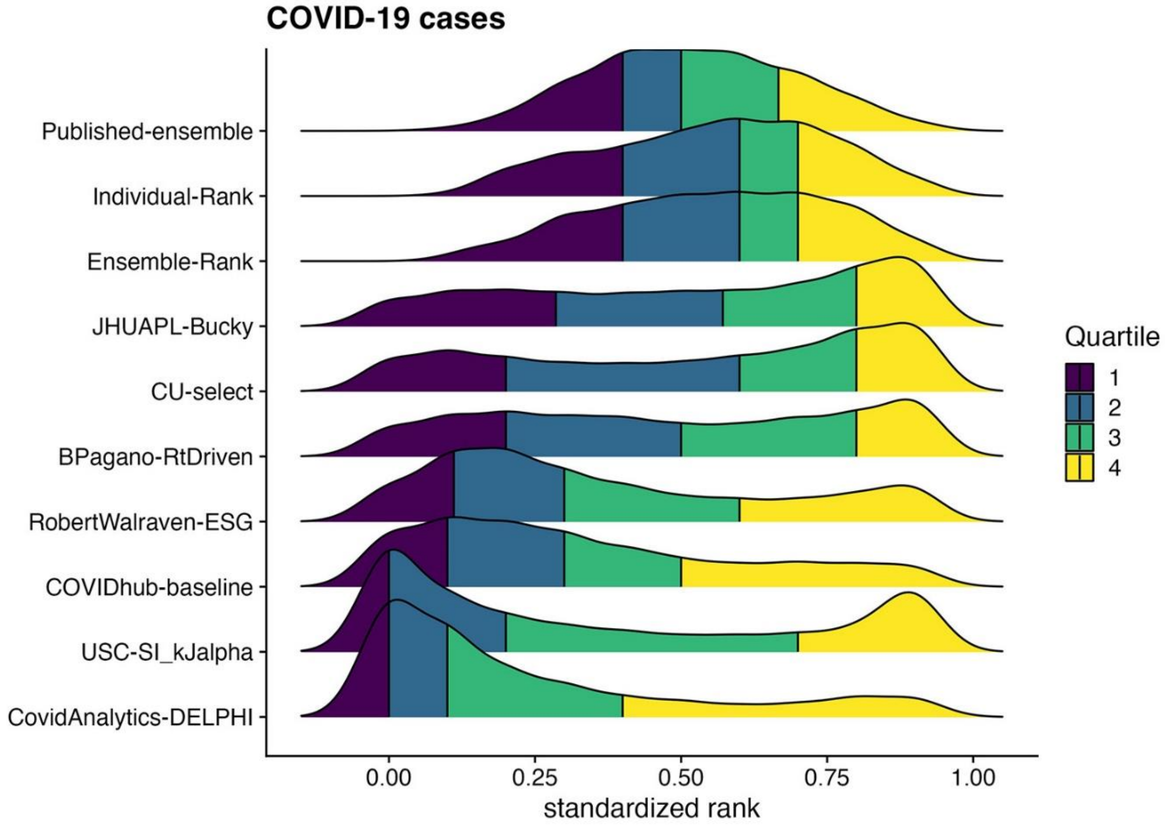

Appendix Figure 4. Distribution of standardized weighted interval score (WIS) rank for forecasts of COVID-19 case counts across every forecasted date, location, and target in the testing period of the analysis. A value of 0 indicates the model had the worst WIS for that particular location, target, and date while a value of 1 indicates that the model had the best WIS. Any density below zero comes from the smoothing of the density plot and should be interpreted as a value of zero. The quartiles of each model's distribution of standardized ranks are shown in different colors: yellow indicates the top quarter of the distribution and purple indicates the bottom quarter of the distribution. The models are ordered by the 25th percentile distribution, with better forecasting models closer to the top. Results for Ensemble and Individual rank models of size four shown.

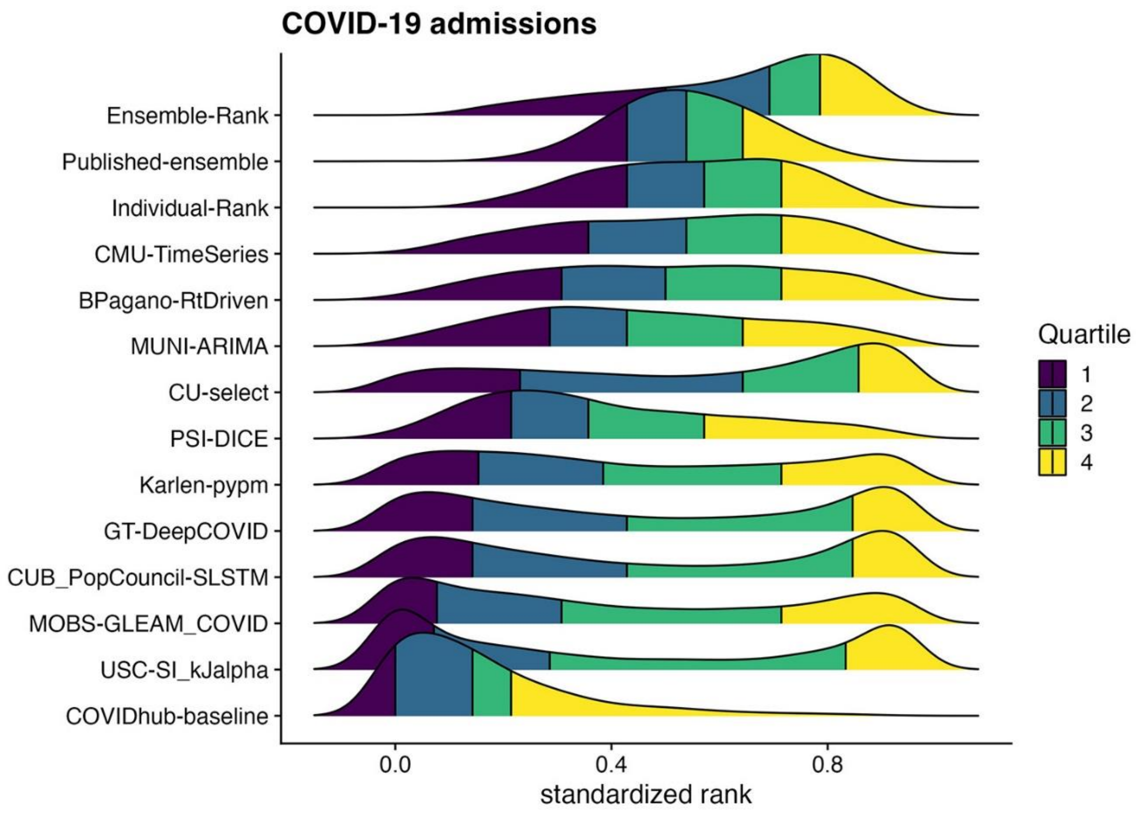

Appendix Figure 5. Distribution of standardized weighted interval score (WIS) rank for forecasts of COVID-19 hospital admissions across every forecasted date, location, and target in the testing period of the analysis. A value of 0 indicates the model had the worst WIS for that particular location, target, and date while a value of 1 indicates that the model had the best WIS. Any density below zero comes from the smoothing of the density plot and should be interpreted as a value of zero. The quartiles of each model's distribution of standardized ranks are shown in different colors: yellow indicates the top quarter of the distribution and purple indicates the bottom quarter of the distribution. The models are ordered by the 25th percentile distribution, with better forecasting models closer to the top. Results for Ensemble and Individual rank models of size four shown.

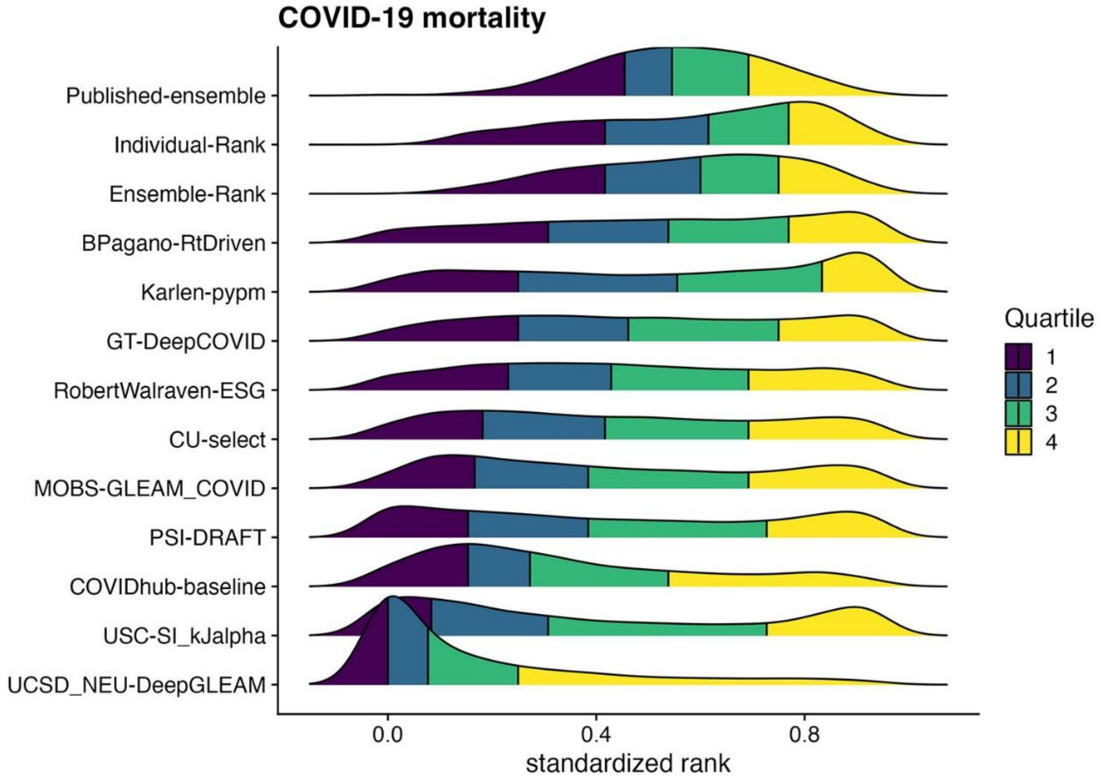

Appendix Figure 6. Distribution of standardized weighted interval score (WIS) rank for forecasts of COVID-19 mortality across every forecasted date, location, and target in the testing period of the analysis. A value of 0 indicates the model had the worst WIS for that particular location, target, and date while a value of 1 indicates that the model had the best WIS. Any density below zero comes from the smoothing of the density plot and should be interpreted as a value of zero. The quartiles of each model's distribution of standardized ranks are shown in different colors: yellow indicates the top quarter of the distribution and purple indicates the bottom quarter of the distribution. The models are ordered by the 25th percentile distribution, with better forecasting models closer to the top. Results for Ensemble and Individual rank models of size four shown.

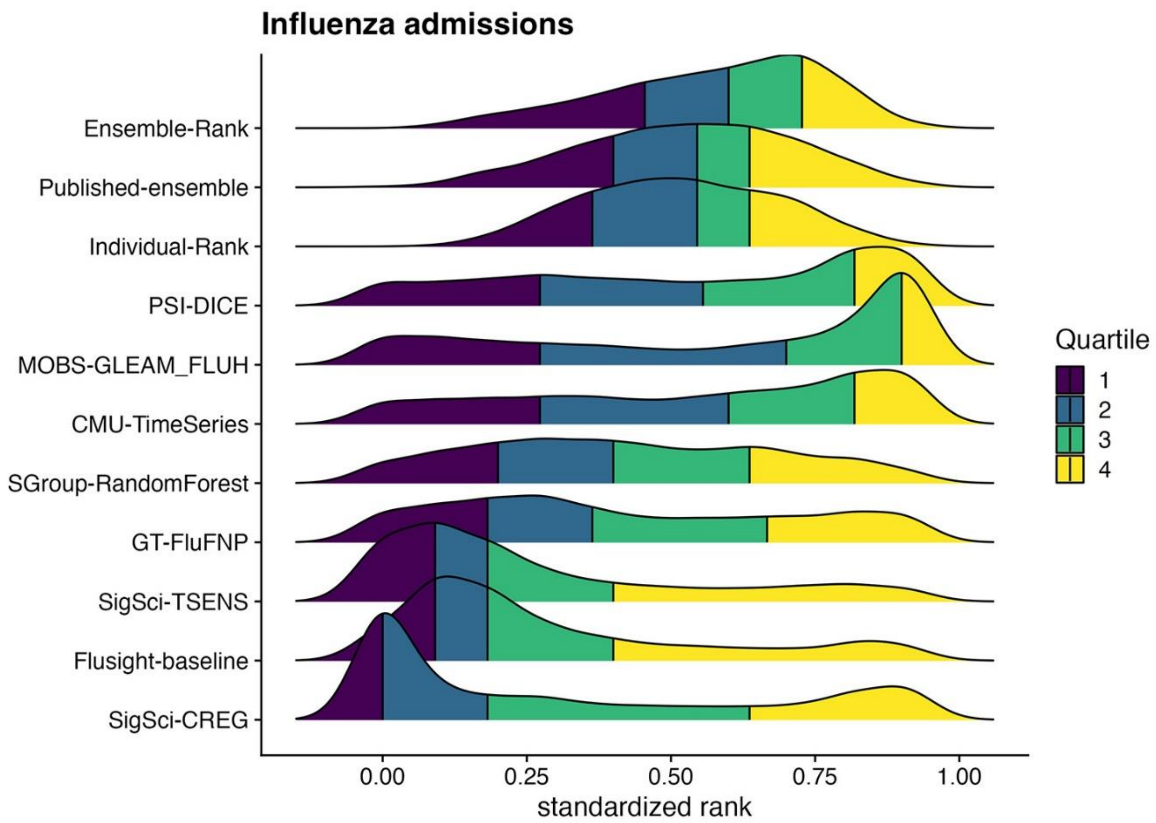

Appendix Figure 7. Distribution of standardized weighted interval score (WIS) rank for forecasts of influenza hospital admissions across every forecasted date, location, and target in the testing period of the analysis. A value of 0 indicates the model had the worst WIS for that particular location, target, and date while a value of 1 indicates that the model had the best WIS. Any density below zero comes from the smoothing of the density plot and should be interpreted as a value of zero. The quartiles of each model's distribution of standardized ranks are shown in different colors: yellow indicates the top quarter of the distribution and purple indicates the bottom quarter of the distribution. The models are ordered by the 25th percentile distribution, with better forecasting models closer to the top. Results for Ensemble and Individual rank models of size four shown.

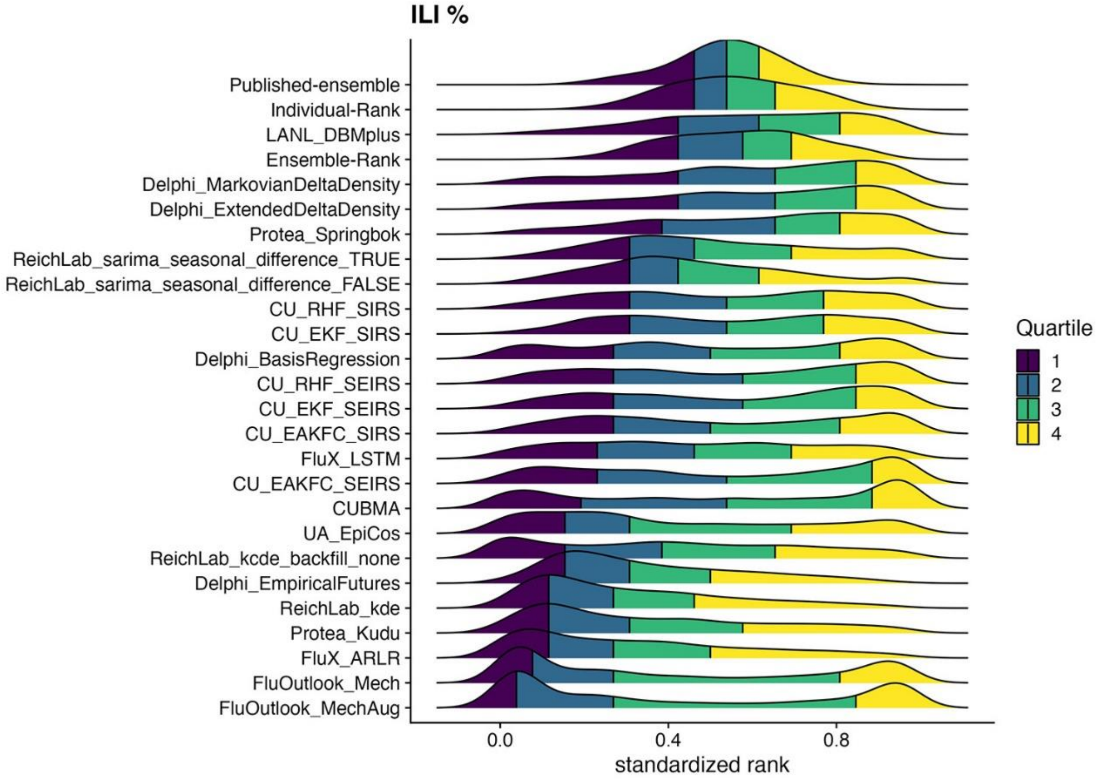

Appendix Figure 8. Distribution of the standardized forecast score rank for forecasts of influenza-like illness (ILI %) across every forecasted date, location, and target in the testing period of the analysis. A value of 0 indicates the model had the worst forecast score for that particular location, target, and date while a value of 1 indicates that the model had the best forecast score. Any density below zero comes from the smoothing of the density plot and should be interpreted as a value of zero. The quartiles of each model's distribution of standardized ranks are shown in different colors: yellow indicates the top quarter of the distribution and purple indicates the bottom quarter of the distribution. The models are ordered by the 25th percentile distribution, with better forecasting models closer to the top. Results for Ensemble and Individual rank models of size four shown.

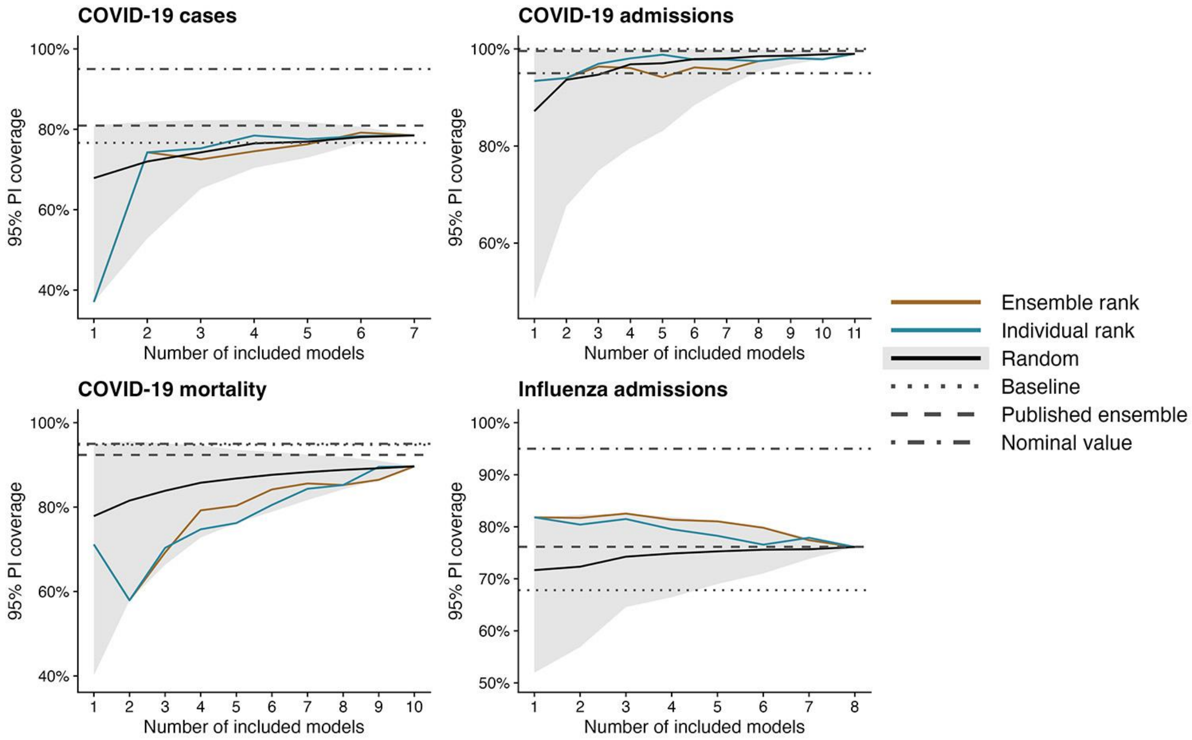

Appendix Figure 9. Forecast prediction interval coverage (PIC) comparison for ensembles of varying ensemble size and component selection strategy on recent influenza and COVID-19 collaborative forecast efforts. Summarized 90% PIC from the recent COVID-19 and influenza forecasting efforts during the respective testing periods across all dates, locations, and forecast horizons (Table S1). Well calibrated models are expected to have PIC near 95% (horizontal dot dash line). We plot the minimum (Grey ribbon, lower), maximum (Grey ribbon, upper), and mean (Solid black line) of random ensemble combinations of a given size (Random), and the trained ensembles composed of the top *n* individual performing models from the training period (Individual rank) or the best performing ensemble of size *n* from the training period (Ensemble rank). We plot the coverage rates from these models alongside the baseline forecast model that makes flat line predictions (horizontal dotted line), and the horizontal dashed line corresponds to the ensemble published in real-time (Published ensemble –horizontal dashed line) that is the unweighted ensemble across all models that submitted for a specific date and forecast target and is used as the gold-standard forecast prediction.

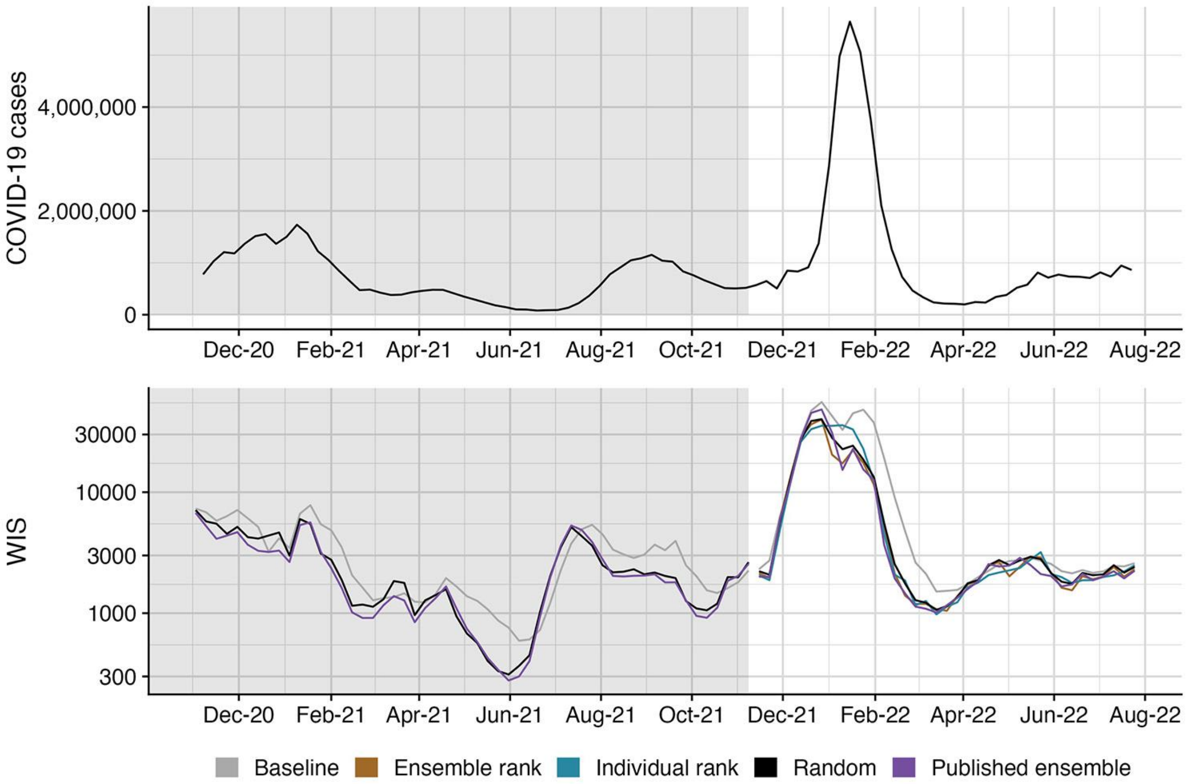

Appendix Figure 10. COVID-19 case counts and model performance by date. (Top) COVID-19 case counts nationally for the United States. (Bottom) Average weighted interval score (WIS) for each model and forecast date across all locations and targets from analysis. Lower scores indicate better forecast performance. Performance is visualized for ensembles of size four for the Ensemble rank, Individual rank, and Random ensembles, and only the mean performance is shown for the Random ensemble. Grey shaded region indicates the training period for ensemble rank and individual rank trained ensembles.

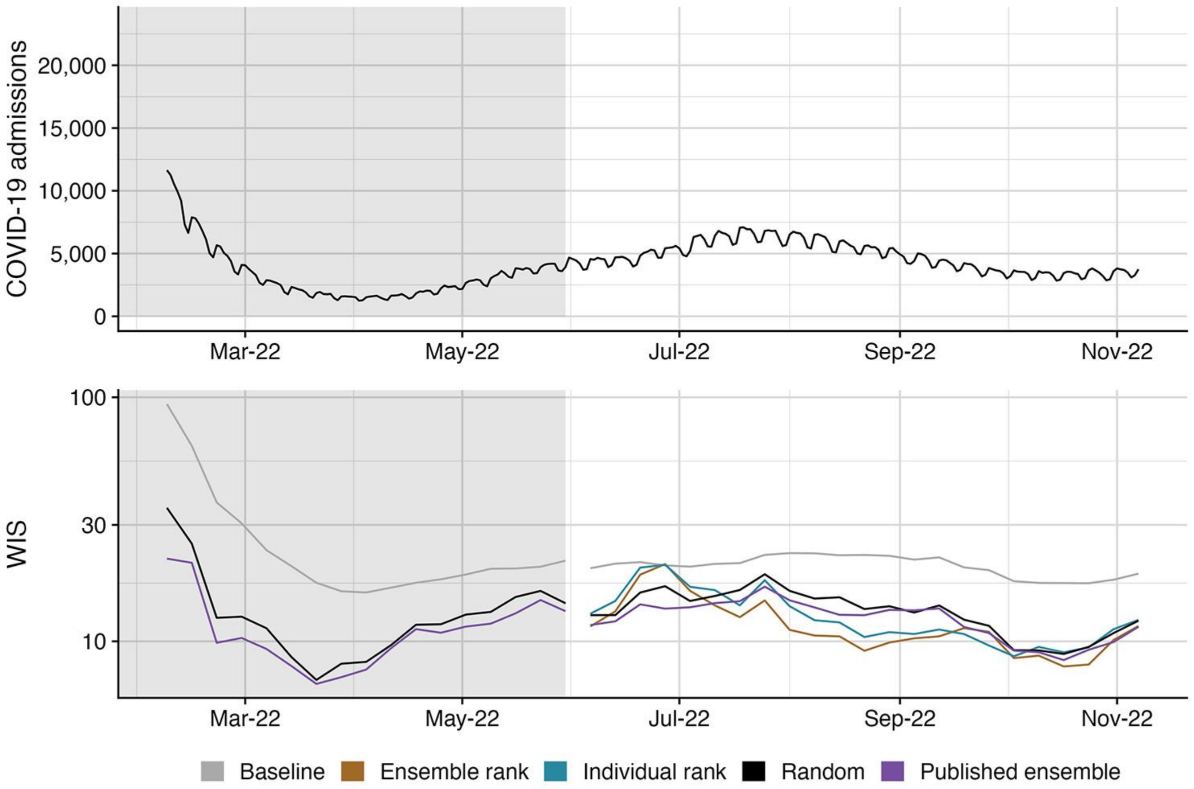

Appendix Figure 11. COVID-19 hospital admissions and model performance by date. (Top) COVID-19 hospital admissions nationally for the United States. (Bottom) Average weighted interval score (WIS) for each model and forecast date across all locations and targets from analysis. Lower scores indicate better forecast performance. Performance is visualized for ensembles of size four for the Ensemble rank, Individual rank, and Random ensembles, and only the mean performance is shown for the Random ensemble. Grey shaded region indicates the training period for ensemble rank and individual rank trained ensembles.

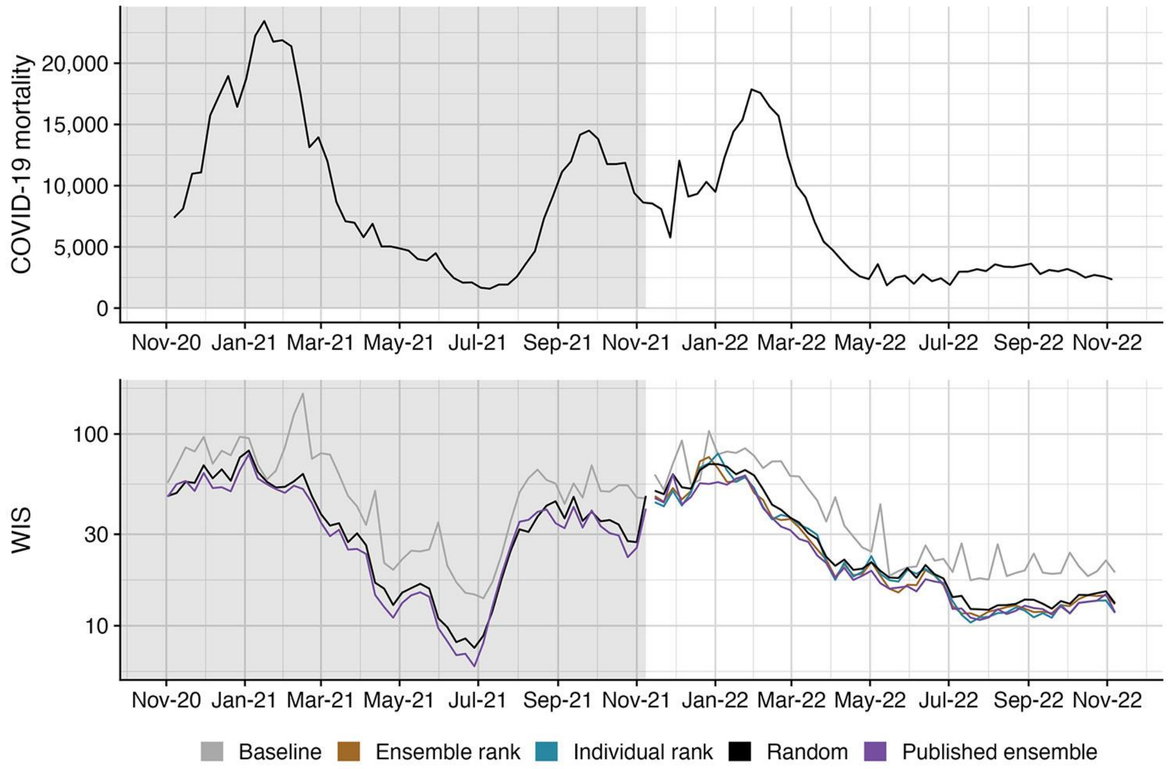

Appendix Figure 12. COVID-19 mortality and model performance by date. (Top) COVID-19 mortality nationally for the United States. (Bottom) Average weighted interval score (WIS) for each model and forecast date across all locations and targets from analysis. Lower scores indicate better forecast performance. Performance is visualized for ensembles of size four for the Ensemble rank, Individual rank, and Random ensembles, and only the mean performance is shown for the Random ensemble. Grey shaded region indicates the training period for ensemble rank and individual rank trained ensembles.

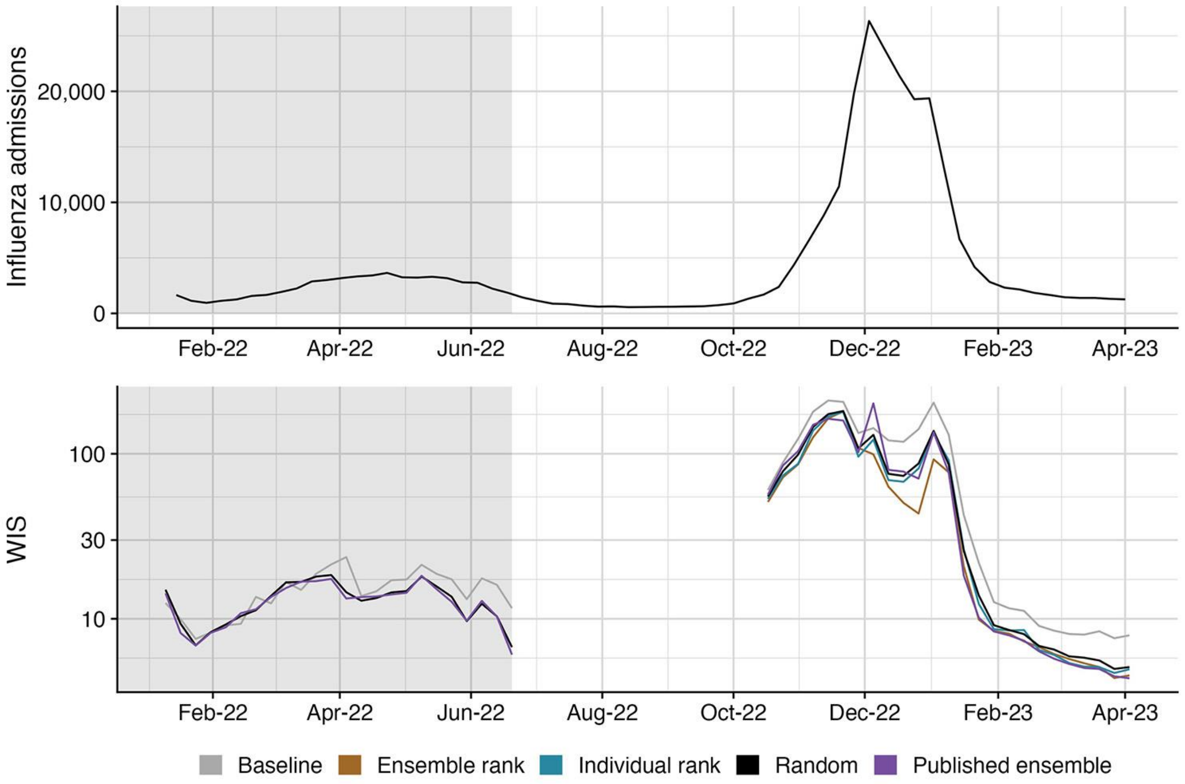

Appendix Figure 13. Influenza hospital admissions and model performance by date. (Top) Influenza hospital admissions nationally for the United States. (Bottom) Average weighted interval score (WIS) for each model and forecast date across all locations and targets from analysis. Lower scores indicate better forecast performance. Grey shaded region indicates the training period for ensemble rank and individual rank trained ensembles. Performance is visualized for ensembles of size four for the Ensemble rank, Individual rank, and Random ensembles, and only the mean performance is shown for the Random ensemble. Performance is not measured during the summer when the collaborative forecast efforts were paused.

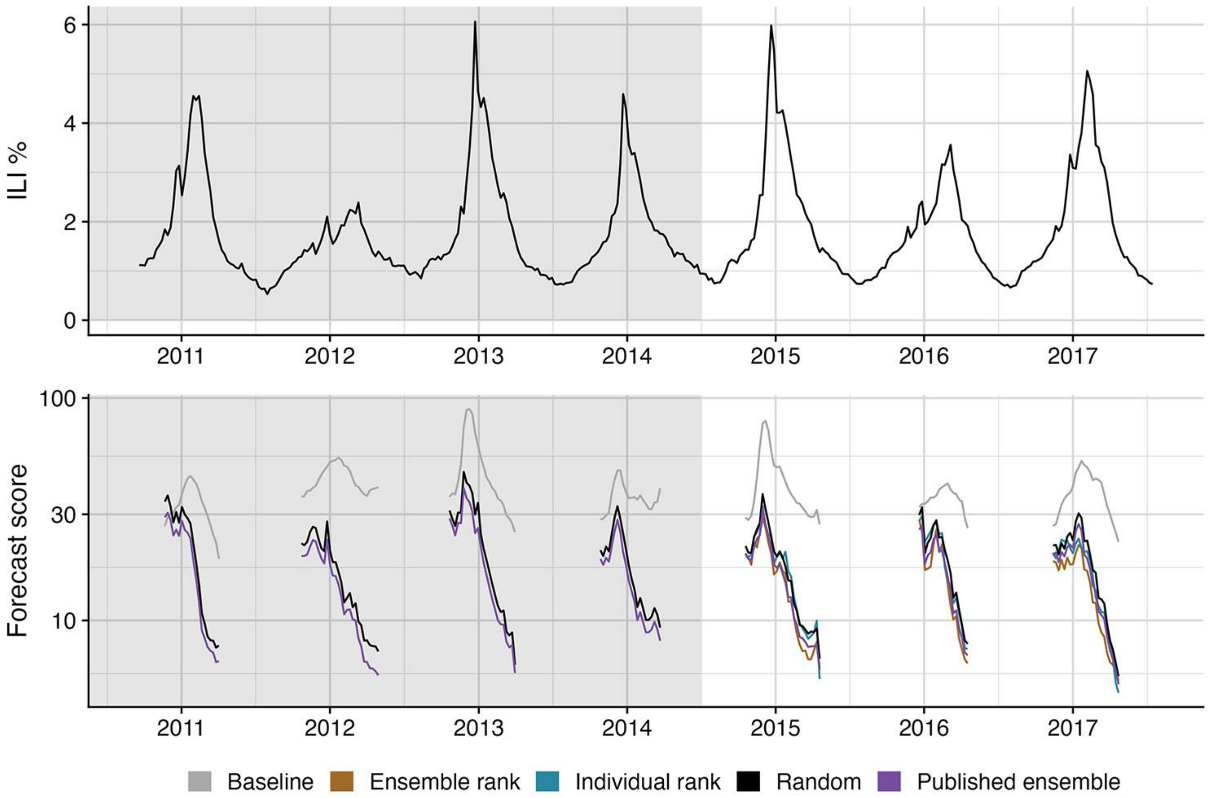

Appendix Figure 14. Influenza-like illness (ILI %) and model performance by date. (Top) ILI % nationally for the United States. (Bottom) Average forecast score for each model and forecast date across all locations and targets from analysis. Lower scores indicate better forecast performance. Grey shaded region indicates the training period for ensemble rank and individual rank trained ensembles. Performance is visualized for ensembles of size four for the Ensemble rank, Individual rank, and Random ensembles, and only the mean performance is shown for the Random ensemble. Performance is not measured during the summer months when the collaborative forecast efforts were paused.

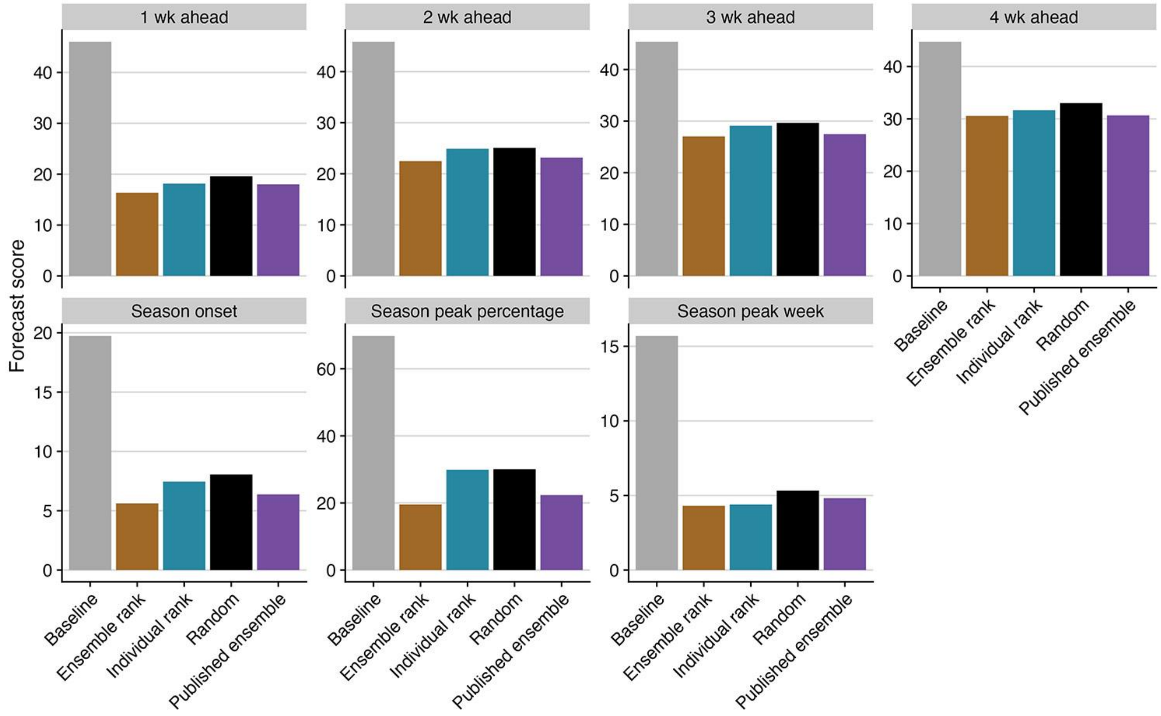

Appendix Figure 15. Influenza-like illness (ILI %) model performance for each forecasted target. Average forecast score for each model and each target (facets) across all forecast dates and locations from the testing period of the analysis. Lower scores indicate better forecast performance. Performance is visualized for ensembles of size four for the Ensemble rank, Individual rank, and Random ensembles, and only the mean performance is shown for the Random ensemble.

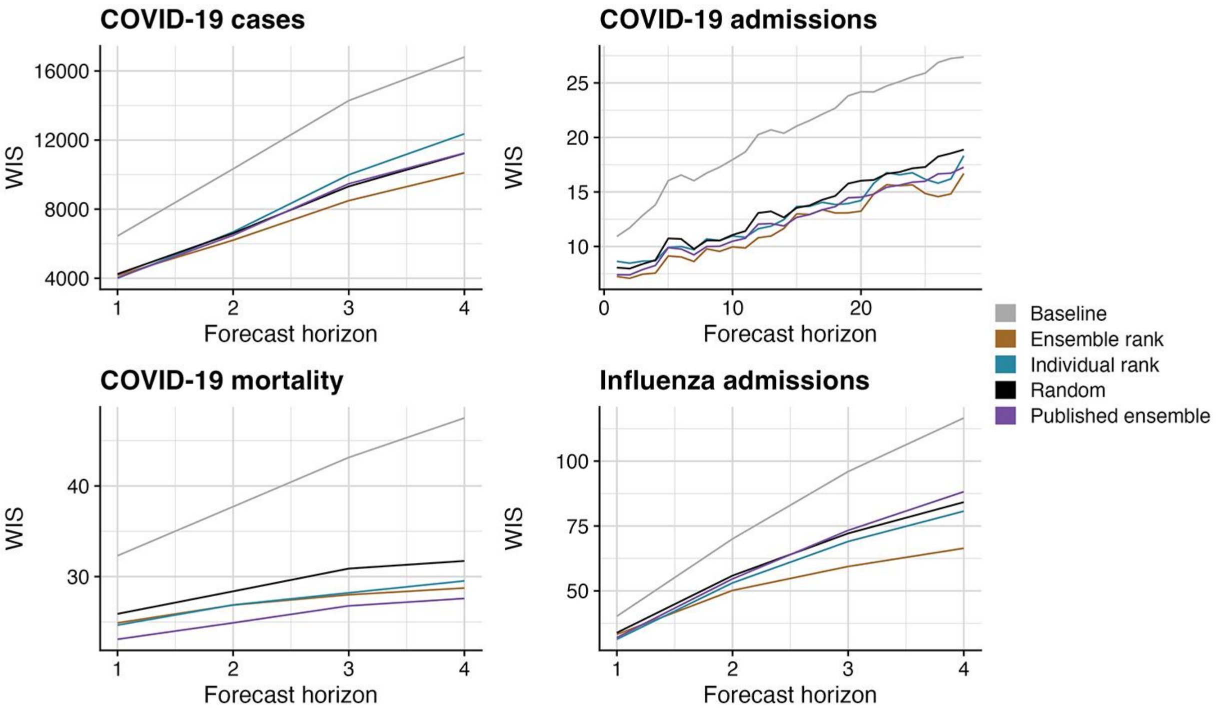

Appendix Figure 16. Model performance for each forecasted target (horizon) across all recent collaborative hub efforts. Average forecast score for each model and each target horizon across all forecast dates and locations from the testing period of the analysis. Lower scores indicate better forecast performance. Performance is visualized for ensembles of size four for the Ensemble rank, Individual rank, and Random ensembles, and only the mean performance is shown for the Random ensemble.

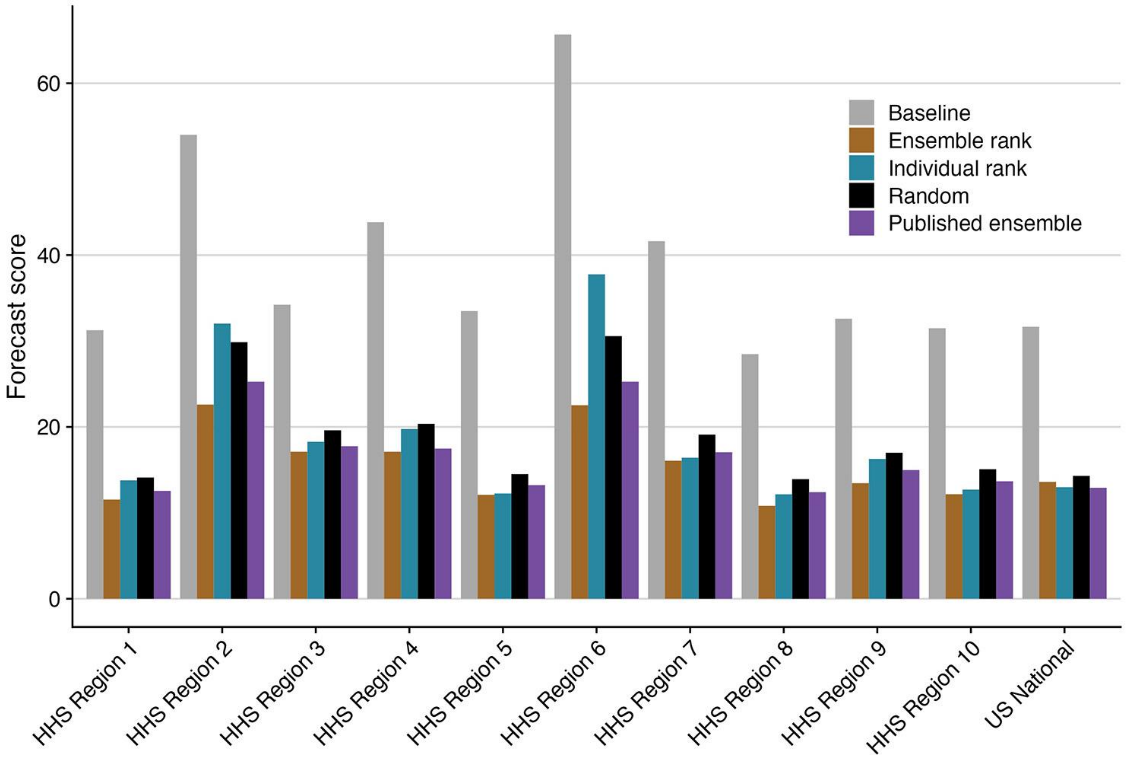

Appendix Figure 17. Influenza-like illness (ILI %) model performance for each forecasted location. Average forecast score for each model and location across all forecast targets and dates from the testing period of the analysis. Lower scores indicate better forecast performance. Performance is visualized for ensembles of size four for the Ensemble rank, Individual rank, and Random ensembles, and only the mean performance is shown for the Random ensemble.

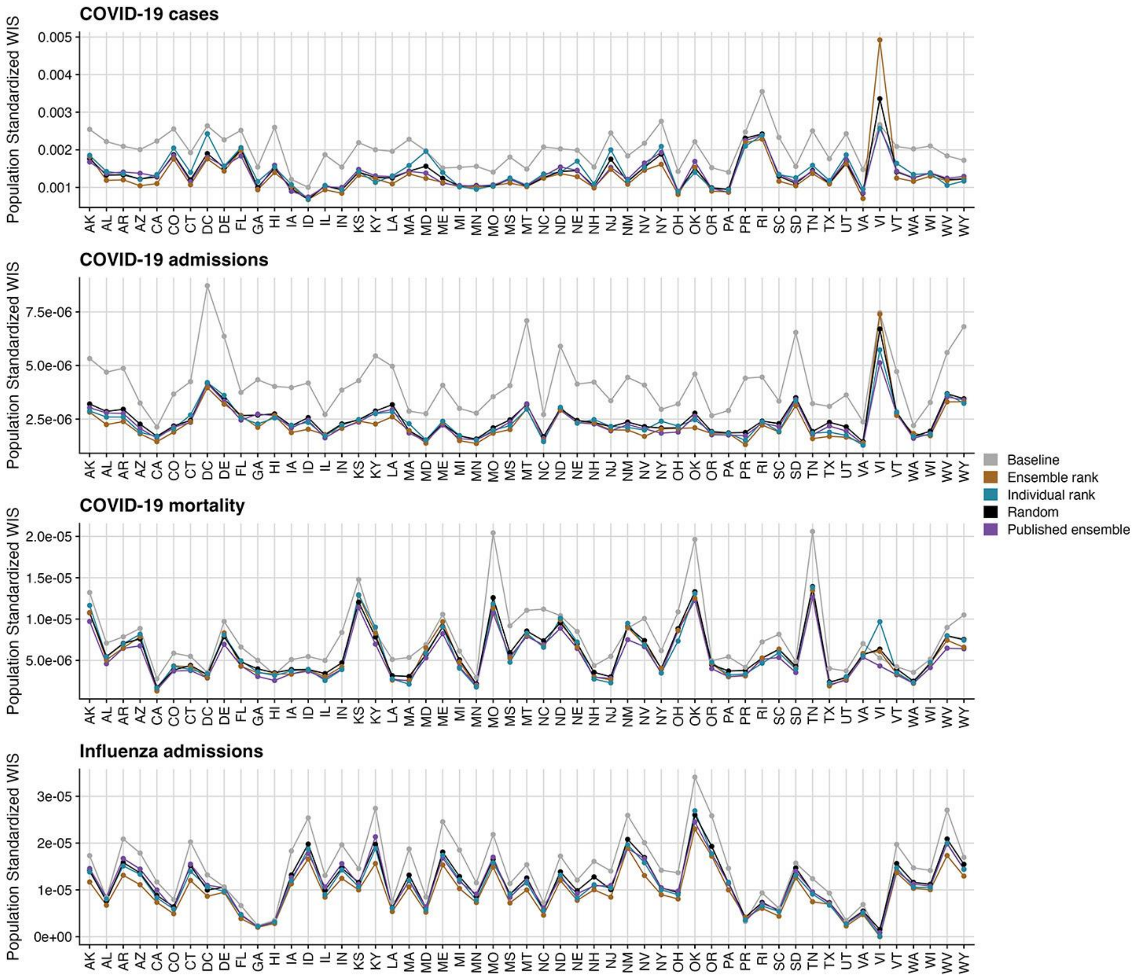

Appendix Figure 18. Model performance for each forecasted location across recent collaborative hub efforts. Average population standardized forecast score for each model and each location across all forecast dates and targets from the testing period of the analysis. WIS was divided by the region's population to account for the absolute nature of the error metric. Lower scores indicate better forecast performance. Performance is visualized for ensembles of size four for the Ensemble rank, Individual rank, and Random ensembles, and only the mean performance is shown for the Random ensemble. Regions are ordered alphabetically by the abbreviation.
